# Statistical modelling of navigational decisions based on intensity versus directionality in *Drosophila* larval phototaxis

**DOI:** 10.1038/s41598-018-29533-0

**Published:** 2018-07-26

**Authors:** Lucia de Andres-Bragado, Christian Mazza, Walter Senn, Simon G. Sprecher

**Affiliations:** 1 Department of Biology, University of Fribourg, Fribourg, Switzerland; 2 Department of Mathematics, University of Fribourg, Fribourg, Switzerland; 3 Department of Physiology, University of Bern, Bern, Switzerland

## Abstract

Organisms use environmental cues for directed navigation. Understanding the basic logic behind navigational decisions critically depends on the complexity of the nervous system. Due to the comparably simple organization of the nervous system of the fruit fly larva, it stands as a powerful model to study decision-making processes that underlie directed navigation. We have quantitatively measured phototaxis in response to well-defined sensory inputs. Subsequently, we have formulated a statistical stochastic model based on biased Markov chains to characterize the behavioural basis of negative phototaxis. Our experiments show that larvae make navigational decisions depending on two independent physical variables: light intensity and its spatial gradient. Furthermore, our statistical model quantifies how larvae balance two potentially-contradictory factors: minimizing exposure to light intensity and at the same time maximizing their distance to the light source. We find that the response to the light field is manifestly non-linear, and saturates above an intensity threshold. The model has been validated against our experimental biological data yielding insight into the strategy that larvae use to achieve their goal with respect to the navigational cue of light, an important piece of information for future work to study the role of the different neuronal components in larval phototaxis.

## Introduction

The nervous system is functionally organized to perceive external cues, which are encoded and decoded to make the correct behavioural decisions. A way of studying the logic of these decision-making mechanisms is through the analysis of robust stereotypical navigational strategies evoked by controlled stimuli (such as light or odour cues) in simple model organisms. Indeed, much of our understanding in the fundamental logic of taxis has been achieved by studying organisms such as *Escherichia coli, Caenorhabditis elegans* or larvae of the fruit fly *Drosophila melanogaster*^1,2^. Navigation in *Drosophila* larvae has been assessed in response to different types of single sensory inputs including vision^3,4^, olfaction^5–8^ and thermo-sensation^9–11^ and a combination of inputs to study multisensory integration^12,13^.

*Drosophila* larvae show robust navigation towards appetitive and away from aversive cues. However, in the absence of an external cue or for a group of blind larvae, the kinematics is essentially random-like^14^. In the presence of a light source, we experimentally show that the taxis is still moderately stochastic, being at most three times less efficient than ballistic kinematics. Therefore, an underlying Markov chain presents itself as an excellent basis for modelling larval kinematics since the external field (light) only introduces a small perturbation that allows a quasi-equilibrium description. That Markov chain is biased to take into account the external cue using Boltzmann’s probabilities^15^. Measurable magnitudes can be obtained by averaging them over the simulated trajectories. Therefore, the properly weighted Markov chain that we introduce has to be understood as a statistical tool to efficiently obtain averaged values of measurable magnitudes. Larval taxis involves a long-term goal, similar to the one discussed by Berman *et al*. for adult flies^16^, that is well described in our model by the intervention of the Metropolis-Hastings algorithm. Stochastic techniques like the one we are proposing here have been recently used to model taxis as a diffusion problem^17^. The power of such stochastic techniques rests on its capacity to tackle complex problems, like diffusion or phase transitions, with a moderate cost in computational time. In particular, the Metropolis-Hastings has been used to efficiently locate global minima of combinatorially-complex objective functions such as the *travelling salesman* problem^18^. In this work, we show how to introduce generalized Metropolis-Hastings weights to bias a Markov chain to extract information from biological experiments where larvae take decisions using information gathered from their immediate surroundings.

Previous approaches to model taxis behaviour have divided the animal’s movements into a set of discrete behavioural states and have analysed the transitions between these states. One modelling approach has been based in a linear non-linear Poisson cascade to model the transition between the larval states^12,13^. Another approach has been to model larval taxis as continuous oscillations whose direction is not controlled by the stimuli but by an *intrinsic oscillator* and where the external stimuli would influence the amplitude of the oscillation^19^.

*Drosophila* larval phototaxis provides an excellent model to study behavioural decision-making because their exposure to the sensory stimulus of light can be tightly controlled^3,4^. Larval paths bear a strong connection with the intensity of light and to the position of the light source, which in the literature has been termed as light directionality^3^. Larvae perceive light through a pair of bilateral eyes, which have been shown to be absolutely essential for visually-guided navigation^4,20–24^. Information from the external field of light is then processed in the brain by a genetically hard-wired decision-making algorithm. Our model allows us to characterize that algorithm as a combination of a goal-directed behaviour layer on top of a stochastic one.

By focusing on the impact of two key excitation elements, light intensity and light directionality, we present a model that studies the interplay between these two components for navigation. We exploit experimental navigational data obtained from various controlled illumination conditions to define probability weights that we use to polarize an underlying Markov chain that has been introduced to analyse larval taxis using stochastic methods. Such a statistical model allows us to simulate larval kinematics with a minimal number of free parameters. Our model provides a theoretical and experimental framework of the decision-making mechanism functioning in the larval brain during navigation.

## Results

### Impact of light intensity and its spatial gradient on taxis

**Figure 1:**
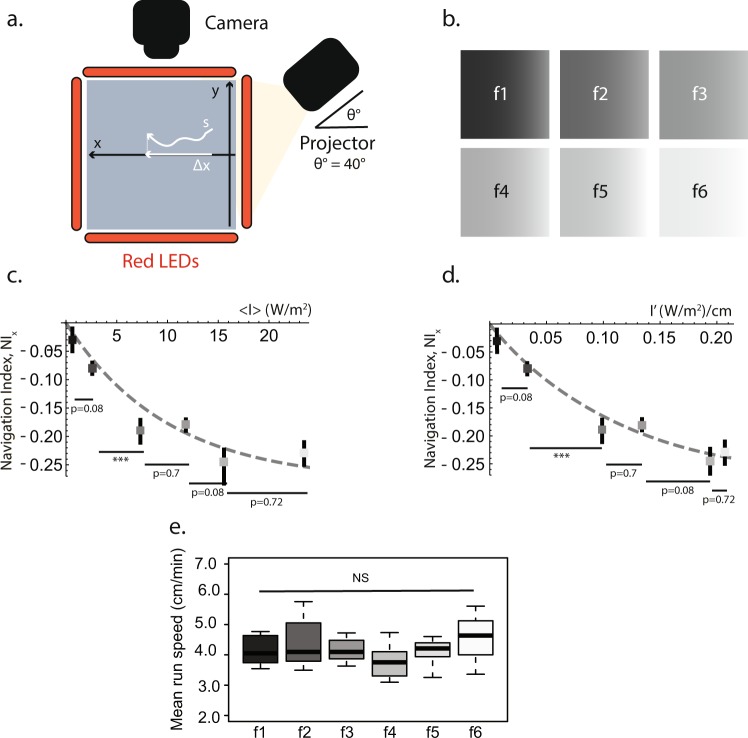
Larval navigation depends on the absolute light intensity and the gradient of the light field. (**a**) Experimental set up formed by the agarose plate (*x* − *y* plane), light source (projector located at *x* = 35.5, *y* = 0, and *z* = 28), forming an angle *θ* = 40° with respect to the agarose plate, and a video-recording camera. The larvae move on the agarose plate and their positions are recorded by the camera. The LEDs placed on the border of the plate aid in the image-acquisition process. The navigation index in the *x* direction (*NI*_*x*_) is calculated by dividing the distance moved in the *x* axis, Δ*x*, by the total length of the stratified path, *s*. (**b**) Filters with different light intensities and gradients, f1–f6 (Supplementary Fig. S1 and Table 1). (**c**) *NI*_*x*_ and standard deviation plotted against the average intensity over the plate for each filter, <*I*> (W/m^2^); the resulting curve shows that the efficiency of larval navigation depends on <*I*>. The dashed line is a least-squares interpolation to data used to guide the eye (see text). The values for the different filters were compared using the Welch t-test and Benjamini-Hochberg was used to correct for multiple comparison. (**d**) Same as (**c**) as a function of the gradient for each filter, *I*′ (W/m^2^/cm). (**e**) Larval mean run speed for the filters f1–f6 takes values from 4.0 cm/min to 4.5 cm/min, which is a statistically non-significant difference (NS). The whiskers of the boxplots represent the range of the mean run speed for the different filters. Within the boxplots for each filter, the middle band is the median (50^*th*^ percentile), and the length of the boxplots shows the 1^st^ and 3^rd^ quartile (25^th^ and 75^th^ percentile).

The navigation index (*NI*) has been used in the literature as a significant statistically-averaged kinematic parameter describing sensory guided navigation^3,4^. Along a given direction (*x*), the *NI* is defined as the mean velocity in that direction, *v*_*x*_, divided by the total velocity in any direction, *v*. In other words, since velocities are measured over a common time interval, the *NI* in a certain direction can be taken as the distance moved in that direction, Δ*x*, divided by the length of the stratified path, *s*. For example, the *NI* along the *x* axis, *NI*_*x*_, would be defined as $N{I}_{x}=\frac{{\rm{\Delta }}x}{s}$ (Fig. 1a). Therefore, the value of the *NI* provides an assessment of the efficiency of larval navigation. In our case, since the source of light was located in the +*x* axis of the agarose plate, the *NI*_*x*_ would be approximately −1 for an object moving ballistically away from the illumination source (negative values for the *NI* imply that larvae navigate towards the −*x* axis). On the other hand, we would obtain a *NI*_*x*_ of approximately 0 for a random walk taken in the absence of an external cue or for blind larvae.

Previous studies have shown that the intensity of light, its spatial gradient and light directionality are relevant factors for visually-guided navigation^3^. Therefore, we hypothesize that these components may be sufficient to explain the observed biological behaviour in our defined experimental framework. First, we experimentally tested the dependence of our primary kinematical measurable magnitude (*NI*) with the two independent variables determined by the external light field: the absolute light intensity and its gradient over the agarose plate where the larvae are located (intensity is measured using irradiance units, as the radiant power flux received per unit area, see Materials and Methods). For this, we used a set of filters where light intensities and their spatial gradients have been varied in a controlled and gradual way: f1, f2, f3, f4, f5 and f6 (Fig. 1b). For all these experiments, the angle of the source of light was kept constant at 40°. Our measurements show that navigation of wildtype larvae depends on the absolute light intensity. Larvae show a very low navigation score when the light intensity is low, such as in f1, where *NI*_*x*_ = −0.03. As the light intensity increases, the value of the *NI* increases in a non-linear way and it saturates for intensities higher than *I* > 20 W/m^2^ to a value below *NI*_*x*_ = −0.3 (Fig. 1c). A heuristic expression that interpolates the dependence of the measured *NI* with the absolute light intensity can be written as:

1 $$N{I}_{x}(I)=-\,0.28(1-{e}^{-I/10})$$

This interpolating function captures the two salient features of this experiment. Firstly, as expected for a kinematical process based on a Markov chain, it approaches zero in the absence of external stimulus (*I* = 0). Secondly, it saturates for around *I* ≈ 20 W/m^2^ (Fig. 1c).

Moreover, larval navigation also depends on the slope of the intensity in the filter, *I*′ (Fig. 1d). Same as for the light intensity, in the absence of a gradient, navigation is quite random-like, *NI* (*I*′ ≈ 0) ≈ 0, and it shows saturation for gradients steeper than *I*′ > 0.20 W/m^2^/cm (Fig. 1d). Again, a heuristic interpolating function that only depends on the gradient of the field of light can be written as:

2 $$N{I}_{x}(I^{\prime} )=-\,0.28(1-{e}^{-10I^{\prime} })$$

The larval speed (about 4 cm/min) is insensitive to different light conditions and shows no correlation to the intensity or the gradient of light (Fig. 1e), being the small variation of velocities statistically non-significant (NS). Therefore, consistently with our use of the *NI*, we dismiss that the saturation of the *NI* could be related to larval velocity.

### Impact of directionality on phototaxis

**Figure 2:**
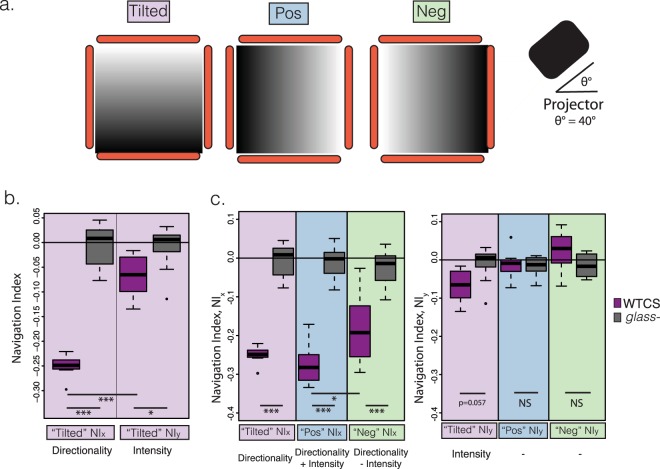
Light directionality plays a big role in larval navigation. (**a**) Projected filters, “Tilted”, “Pos” and “Neg”, used to study the joint effect of light intensity and light directionality. All these filters were projected individually in the agarose plate and the light source was always in the +*x* side as shown in Fig. 1. Light intensity varied differently in all of them: in “Tilted” it increased along the the +*y* axis, in “Neg” it increased along the −*x* axis and in “Pos” it decreased along the −*x* axis. (**b**) Wild-type Canton S (WTCS) larvae (dark purple boxplot) in the “Tilted” filter have a statistically-significantly different navigation index both in the *x* direction (*NI*_*x*_ = −0.25, *p* > 0.001) and in the *y* direction (*NI*_*y*_ = −0.07, *p* = 0.0152) compared with the effectively blind *glass* mutants (grey boxplot) navigating in the same filter. The directionality effect (measured in “Tilted” by *NI*_*x*_) is stronger than the light intensity effect (measured by *NI*_*y*_) as most of the larval navigation is in the *x* axis (*p* < 0.001). (**c**) Navigation index in the *x* axis (*NI*_*x*_) (left graph) and in the *y* axis (*NI*_*y*_) (right graph) for the filters showed in (A) (“Tilted” in pink, “Pos” in blue and “Neg” in green) for both WTCS (dark purple) and the blind *glass* mutant larvae (grey). Larvae presented with the “Pos” filter have the highest navigation index (*NI*_*x*_ = −0.27), as both the light intensity and light directionality drive larvae to navigate in the −*x* direction. Larvae navigating in the “Neg” filter have a lower navigation index (*NI*_*x*_ = −0.18) as both effects drive them to navigate in opposing directions (light intensity towards +*NI*_*x*_ and light directionality towards −*NI*_*x*_). The range of *NIs* is given by the whiskers of the boxplots. The median (50^*th*^ percentile) is represented with the middle line and the 25^th^ and 75^th^ percentile are represented by the lower and top bands of the boxplot respectively. p-values are calculated with the Welch’s t-test and using the Benjamini-Hochberg procedure to correct for multiple comparison, where *p* < 0.001 is represented by ***, *p* < 0.01 ** and *p* < 0.05 by *.

In a natural environment, light emitted from an external cue harbours directional as well as intensity information^3^. To test the relative importance of these two components, we have projected a light pattern labelled as “Tilted” (Fig. 2a) in which the intensity linearly decreases along the *y*-axis, thus perpendicular to the light directionality along the *x*-axis. Contrary to the series of filters f1-f6, where both the light intensity and directionality drive larvae towards the same direction (−*x* axis), in the “Tilted” pattern, both effects are decoupled into two components: the light intensity artificially decreases in the −*y* direction because of the projected filter, while at the same time it naturally decreases along the −*x* direction as an effect of the increasing distance to the projector (Supplementary Fig. S3). Consequently, in the “Tilted” pattern, *NI*_*y*_ mainly accounts for larval navigation due to the variation of the light intensity, while *NI*_*x*_ could be taken as a proxy of the larval navigation away from the light source. We next quantified larval navigation along the *x*− and *y*-axis independently (Fig. 2b). Wildtype larval phototaxis can be explained both by the light source avoidance (Fig. 2b, “Tilted” *NI*_*x*_) and by the avoidance of higher light intensities (Fig. 2b, “Tilted” *NI*_*y*_). However, we find a significant difference. Negative phototaxis in the “Tilted” pattern is driven along the x-axis, where light directionality is most important, more than three times higher (*NI*_*x*_ = −0.25) than along the y-axis, (*NI*_*y*_ = −0.07), which is mostly related to intensity. Since the latter value is quite small, we stablish its significance by comparing with the *NI*_*y*_ of visually-blind control *glass*^*j60*^ homozygous mutant larvae, which completely lack eyes (*NI*_*y*_ *glass*^*j*60^ = −0.01, *p*–*value* = 0.015). This proves that even if the wildtype *NI*_*y*_ for “Tilted” is small, it still remains light-intensity-driven taxis. Moreover, as expected, the *NI*_*x*_ for wildtype larval navigation in this “Tilted” pattern is also statistically-significantly different from the *NI*_*x*_ navigation of blind larvae (*NI*_*x*_ *glass*^*j*60^ = −0.003, *p* < 0.001). Therefore, we conclude that both the *NI*_*x*_ and the *NI*_*y*_ navigation of wildtype larvae in the “Tilted” pattern are due to the visual system and not to other effects.

To further investigate the relationship between directionality and light intensity, we have generated a pattern labelled as “Pos” (for positive) where both effects reinforce each other in the same direction and another one where they compete in opposite directions along the −*x* and +*x* axis, labeled as “Neg” (for negative) (Fig. 2a). We observed that navigation is stronger for reinforcing intensity and directionality cues (“Pos”, *NI*_*x*_ = −0.27) than for competing ones (“Neg”, *NI*_*x*_ = −0.18). This also supports our finding from the “Tilted” pattern that the effect of light intensity is weaker than the directional one, since the difference in navigation indexes between “Pos” and “Neg” is just −0.09 (Fig. 2c and Table 1), which we interpret as the drive towards +*x* in “Neg” (intensity) only subtracting about one third of the drive towards −*x* in “Pos” (directionality and intensity). Furthermore, the effectively blind *glass*^*j60*^ mutant larvae have a *NI* that is statistically indistinguishable from zero (Fig. 2b,c), which proves that besides the different patterns of light, all other conditions are kept the same in all these three experiments.

**Table 1:** Experimental and simulated navigation indexes for all the projected patterns of lights used in the experiments (f1–f6, “Pos”, “Neg” and “Tilted”).

Filter	*NI*_*x*_	*NI*_*y*_	*ni*_*x*_	*ni*_*y*_	*T*
f1	−0.03 ± 0.02	−0.03 ± 0.01	−0.03 ± 0.004	−0.001 ± 0.005	4.93
f2	−0.08 ± 0.01	−0.03 ± 0.01	−0.08 ± 0.01	−0.002 ± 0.01	8.18
f3	−0.19 ± 0.02	0.02 ± 0.01	−0.19 ± 0.01	0.003 ± 0.01	10.06
f4	−0.18 ± 0.01	−0.01 ± 0.01	−0.18 ± 0.01	−0.003 ± 0.01	19.37
f5	−0.25 ± 0.03	0.01 ± 0.01	−0.25 ± 0.01	0.002 ± 0.01	16.51
f6	−0.23 ± 0.02	0.01 ± 0.02	−0.23 ± 0.01	0.003 ± 0.02	26.51
“Pos”	−0.27 ± 0.02	−0.016 ± 0.01	−0.27 ± 0.01	−0.001 ± 0.01	10.95
“Neg”	−0.18 ± 0.03	0.02 ± 0.01	−0.18 ± 0.01	0.001 ± 0.01	13.8
“Tilted”	−0.25 ± 0.01	−0.07 ± 0.02	−0.25 ± 0.01	−0.07 ± 0.01	8.75
D90-f1	0.12 ± 0.04	0.03 ± 0.04	0.12 ± 0.01	0.00 ± 0.01	9.5
D90-f2	0.14 ± 0.05	0.01 ± 0.06	0.14 ± 0.01	0.00 ± 0.01	7.0
D90-f3	0.09 ± 0.04	−0.01 ± 0.04	0.09 ± 0.01	0.00 ± 0.01	9.5

Experimental navigation indexes (dimensionless) in *x* (*NI*_*x*_) and *y* (*NI*_*y*_) directions, and the corresponding simulated navigational indexes, *ni*_*x*_ and *ni*_*y*_ for a given effective temperature *T* (W/m^2^) using $f(\alpha )=1-{(\frac{\alpha }{180})}^{4}$. The standard deviation of the experimental *NI* was calculated for 10 experiments for each illumination condition with around 30 larvae each. The standard deviation for the simulated *ni* was calculated with 30 simulations for each case.

**Figure 3:**
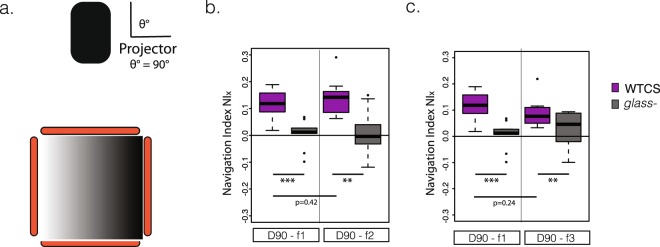
Larval navigation in the absence of directionality. (**a**) Source of light (projector) is located in a zenithal position (θ = 90°) (**b**) D90-f1 compared with D90-f2: different absolute intensities but same gradient result in similar NI (**c**) D90-f1 compared with D90-f3: similar absolute intensities but different gradients affect the navigation index in agreement with the prediction of the proposed model.

We also explored larval navigation under conditions where the directionality factor is minimal. For this purpose, we set a high-intensity projector forming an angle *θ* = 90° with the agarose plate (Fig. 3a).

We projected three different patterns. A pair of them compared a similar intensity gradient, but a different value of the average intensity (D90-f1 and D90-f2, Supplementary Fig. S5 and Supplementary Table S6). The other pair has a similar average intensity, but significantly different gradients (D90-f1 and D90-f3, Supplementary Fig. S5 and Supplementary Table S6).

In all these three filters, the WTCS larvae showed a statistically-significant navigation towards the darker areas of the agarose plate in comparison with the blind glass mutant larvae.

The difference between the larval navigation for the two filters which have the same slope is small (NIx = 0.12 for D90-f1 and NIx = 0.14 for D90-f2) and statistically not significantly-different from each other (p-value = 0.42, Fig. 3b). The difference in larval navigation in the two filters that have a different slope is larger (D90-f1, NIx = 0.12 and D90-f3, NIx = 0.09). Even if the difference is not statistically significant (p-value = 0.24, Fig. 3c), a slight tendency can be seen where larvae have a more efficient navigation when exposed to a steeper light gradient (D90-f1, NIx = 0.12) than one that is not so steep (D90-f3, NIx = 0.09). This agrees with our model that predicts that without directionality, the gradient of light is the main driving force for taxis.

Table 1 provides values for experimental and simulated navigation indexes for all the projected filters.

### Simulation of taxis as a function of intensity and directionality

Next, we propose a statistical model for larval phototaxis that allows us to rationalize the experimental results presented above in a unified way. Such a model has been built taking into account the following two significant facts: (i) in the absence of light the observed kinematics is well described by a Markov chain resulting in a random walk characterized by *NI* = 0, and (ii) even for the higher intensities, the *NI* takes relatively low values, indicating that the external field amounts to a non-negligible but small perturbation on the kinematics. Dealing with a perturbation has the distinctive advantage that we may assume quasi-equilibrium, same as in a diffusive regime on a physical system^25^. Under these conditions, we start building a Markov chain that reproduces well the kinematics in the absence of a field of light, and we describe the perturbation caused by the external field by biasing the Markov chain using weights that are appropriate to describe the experiments. This is achieved by imposing simple biological considerations operating on the larvae; in a transition between states ***r*** and ***r***′ in the Markov chain, we define the following weights:

3 $$W({\boldsymbol{r}}\to {\boldsymbol{r}}^{\prime} )={\rm{\Delta }}I({\boldsymbol{r}}\to {\boldsymbol{r}}^{\prime} )+\beta  < \,I\, > f(\alpha ({\boldsymbol{r}}\to {\boldsymbol{r}}^{\prime} )),$$

with

4 $$f(\alpha )=1-{(\frac{\alpha }{180})}^{4}$$

**Figure 4:**
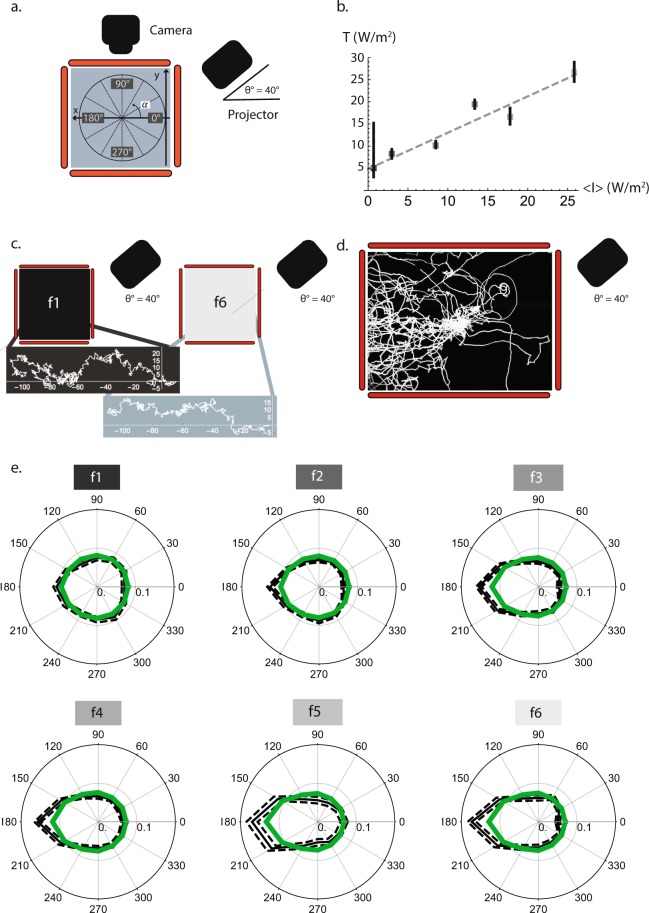
The model yields simulated larvae with similar navigation characteristics to the experimental larvae. (**a**) Coordinate axis used within the agarose plate to define larval movement. The angle *α* was defined within the *x* − *y* plane of the agarose plate with respect to the *x* axis, where 0° and 180° are the direction towards and away from the light source respectively. (**b**) The effective temperature *T* (W/m^2^) increases proportionally to the average intensity <*I*> (W/m^2^) of the light field. The dashed line is a linear interpolation to guide the eye. The error bars for the effective *T* have been calculated using the experimental error for the navigation index (*NI*) and calculating the *T* for *NI* plus and minus this error. (**c**) Simulated larval paths for f1 and f6. Each path shows one simulated larva, which starts at (0, 0) and moves towards the −*x* side of the plate, avoiding the projector which is located on the +*x* side of the agarose plate (right hand side). The model yields simulated larval paths reflecting the stochastic underlying Markov chain, but also targeted navigation to get away from the light source and from the regions of high light intensities. Taxis is more targeted for higher intensity conditions (f6) same as observed in the experiments. (**d**) Experimental larval paths for the f6 filter (30 experimental larvae are shown); these paths are similar to the simulated ones seen in (**c**). (**e**) Comparison of the relative probability of orientation with respect to the light source (located at the right, at 0°) both for simulated (green) and experimental (grey) larvae for the different light conditions (f1, f2, f3, f4, f5 and f6). Larvae are oriented at 180° when they navigate away from the light source, and at 0° when they navigate towards the light source. For the analysis, both the experimental and the simulated larval angular probability distributions, *P*(*α*), were binned in 30°. The simulated larvae have been calculated using $f(\alpha )=1-{(\frac{\alpha }{180})}^{4}$. Error bars are shown for the experimental angular distributions and they were obtained from the Matlab output files from the MAGAT Analyzer.

The first term of equation (3) gives the difference in intensity experienced by larvae while taking a step from position ***r*** to ***r***′, which depends on the gradient of the intensity, Δ*I* = *I*(***r***) − *I*(***r***′). The second term is meant to describe the directional factor. This term is proportional to the average intensity <*I*> (in units of irradiance, W/m^2^) for each projected pattern, as it deals with the increased reaction of larvae against brighter sources of light, which is hinted by the experimental results in Fig. 1c. It carries an angular dependence through the function *f* of the direction *α* in the transition ***r*** → ***r***′ (Fig. 4a). We have tried different models for *f* (*α*) that will be discussed below (Materials and Methods, Determination of *f* (*α*)) and eq. (4) shows the one that yields a best fit to the experimental angular distribution probabilities. Finally, *β* is a free parameter that allows us to balance the unknown relative importance between directionality and intensity gradient according to the experimental evidence. This parameter is obtained from a specifically designed pattern of light (“Tilted”, Fig. 2a), where the first term dominates the *NI* in one direction, while the second term dominates the *NI* in a perpendicular direction. Such a pattern provides two nearly independent experimental values for the *NI* that can be used to determine the relative importance of the two terms in eq. (3). For the case the projector was used forming a 90° angle with the agarose plate, we can write *β* = *β*′ ∗ cos *θ*, which takes into account the projection of the source of light (projector) on the larval eyes. Notice that eq. (3) is used inside an iterator that propagates the simulated larvae from the origin to the edge of the plate through a succession of steps going from r(t_1_) to r′(t_2_), t_2_ > t_1_.

Starting from a state ***r***, the probability of accepting the new state ***r***′ is then defined by the factor, ${e}^{-W({\boldsymbol{r}}\to {\boldsymbol{r}}^{\prime} )/T}$ (Materials and Methods, generalized Metropolis-Hastings), where *T* is a parameter that, following a thermodynamics simile, plays the role of an effective temperature (measured in the same units as *W*, W/m^2^). We find that the larval effective temperature has higher values for more intense light fields (f6 compared to f1, Fig. 4b). We interpret the consequences of this behaviour in the Discussion section. The error bar for *T* corresponding to the darkest filter (f1) in Fig. 3b is larger than for the other cases because in the absence of light, the larval movement ceases to be targeted and becomes similar to a random walk, where *T* is not meaningful anymore and cannot be determined. In statistical terms, the effective temperature is obtained from a probability that depends on the quotient $\frac{W({\boldsymbol{r}}\to {\boldsymbol{r}}^{\prime} )}{T}$. In the absence of an external field we have *W* = 0, and any value of *T* corresponds to the same probability so its value is not well defined (unbiased Markov chain).

The biased Markov chain is not time reversible (principle of detailed balance) since it is driven by an external field that imposes a definitive direction over time. However, in a similar process to the equilibration pattern followed by a thermodynamics system, we have found both in our simulations and in our experiments that important kinematical indicators, like the *NI*, reach a steady state after some initial fluctuating steps. Therefore, these indicators converge to a well-defined value and can be safely compared between simulations and experiments.

The angular part in *W*, *f* (*α*), is a dimensionless function chosen to obtain the best possible fit to the experimental angular probability distributions. We have found that a good choice for *f* (*α*) is to make it proportional to a power of the angle *α*^*n*^, where *α* is the angle formed in the plane of the agarose plate between the attempted direction ***r***′ and the *x* axis (Fig. 4a), and the power *n* can be taken as a free parameter that is chosen to obtain the best fit to experiments. In particular, we have found that *n* = 4 is an optimal value. Possible choices for *f* (*α*) are described in more detail below (Materials and Methods, Determination of *f* (*α*)).

Our statistical model yields more targeted paths when the intensity and its gradient are higher (Fig. 4c, f6 compared to f1) which leads to higher values for *NI*. Furthermore, we notice that simulated larval paths look similar to the experimental ones (Fig. 4d) although such a similarity cannot be pushed too far since it cannot be precisely quantified. On the other hand, the similarity of the angular distribution for the experimental larvae and the simulated ones proves that our model quantitatively reproduces experimental results under different light conditions. Figure 4e shows the agreement between experimental (grey curves) and simulated (green curves) angular distributions obtained with the non-linear $f(\alpha )=1-{(\frac{\alpha }{180})}^{4}$ for filters f1 to f6.

## Discussion

### Larvae respond differently to different intensities and light gradients

Our results show that larval navigation depends on light intensity and its gradient and, that larvae navigate more efficiently (larger *NI*) when the light intensity is higher and when the gradient of the intensity is steeper. Furthermore, this non-linear behaviour (Eqs (1) and (2)) saturates for intensities higher than *I* > 20 W/m^2^ (Fig. 1c) and for intensity gradients higher than *I*′ > 0.2 W/m^2^/cm (Fig. 1d). As commented above, we do not assign such a saturation behaviour to a lack of physical response from the larvae since the mean velocity is nearly independent of the illumination conditions, but rather to a limited capacity for processing information in the underlying neural network in the larval brain. From a biological point of view, larvae make decisions to take a step from position ***r*** to ***r***′ or not, based on the *local* conditions surrounding them. These conditions are the only ones that larvae can probe with their limited visual organ and the only ones that they can process in their brains without requiring an expensive memory process to record a string of magnitudes all along their paths. Saturation regarding the light intensity can be understood from a limited ability to process the input signal of too many photons. On the other hand, the experimental evidence that larvae navigate differently depending on the gradient of the intensity implies that larvae must read the gradient of the field of light (*I*′). Such an operation needs to measure the intensity in two close points and then proceed to compare them. Therefore, it involves a *memory* process if it is to be done at two subsequent times along the larval path.

Similarly, in the model, whether a transition in the Markov chain happens or not is based on variables that can be locally obtained using only the larval starting and intended positions.

### Distinct larval directional tendency to move away from the light source

The combination of our experiments with our statistical model shows the detailed relationship between light intensity and directionality for larval phototaxis for the first time. The analysis of the “Tilted” pattern by our statistical model tells us that the main reason for larval phototaxis, in about a one to three ratio, is to get away from the source of light, rather than to simply move to darker regions. This is in part due to the saturation process shown in Fig. 1c,d, that limits the effect of the first part in eq. (3). The weights defined in our model show, in a quantitative way, that larvae can process the relative orientation of light. Such a capacity to discriminate different angles explains the larval ability to move maximizing the distance to the source of light by simply giving a higher probability to angles around 180° (away from the light source) than to angles around 0° (towards the light source).

We have also shown that larvae still navigate in the absence of directionality (Fig. 3) and that the difference in navigation in the absence of directionality seems more triggered by a different light intensity slope rather than by absolute intensity. The fact that under these conditions the slope is more important for larval navigation than the absolute light intensity agrees with the prediction of our model.

### Larval phototaxis and the proposed statistical model

An interesting feature of our statistical model is that it only requires three adjustable parameters: the relative balance between intensity and directionality *β*, eq. (3); the power *n* telling how sensitive the larvae is to changes in the light direction, eq. (4); and the effective temperature *T* that determines the stochastic exploration (see eq. (5) below). The reason for needing so few parameters is probably linked to the general principles governing the generalized Metropolis-Hastings algorithm, which takes care of the statistical behaviour in a way that is known to work well for many different complex systems found in nature. The model is based in principles so well accepted in different contexts that except for particular details in eq. (3), it should work for other organisms and other sensory cues.

The related Metropolis-Hastings algorithm has been successfully used to efficiently locate *global* minima of combinatorially-complex objective functions such as the travelling salesman problem^18^. In contrast, we remark that our biological experiments mostly bring information about larval decisions taking into account *local* data (intensities and gradients in the immediate surroundings of the organism) and proceed with a limited amount of neural circuitry. Therefore, the weights governing the simulation in eq. (3) should be interpreted more as a local solution to the problem rather than a global one.

The value of the effective *T* in the simulations is adjusted so that the currents of larvae going towards the light source and in the opposite direction match the experimental *NI* measured under some particular light conditions. Therefore, this *T* determines a quasi-equilibrium condition, similar to the one found in a chemical reaction where reactants convert into products, and vice versa, in ratios that match the actual production at a given temperature. On the other hand, such a parameter lends itself to a biological interpretation; it controls the larval probability of taking risks by either going to higher intensity regions or by getting closer to the source of light. Such a behaviour is known to be a useful way to avoid being trapped in local minima, as it has been proved when simulated annealing has been applied to find the global minimum of a given objective function. Our results show that the effective temperature grows with light intensity and its gradient. These are conditions that from a biological point of view should require more vigorous action from the organism to quickly find a more convenient position. In turn, when conditions are not so harsh, organisms prefer taking conservative decisions, hardly moving to worse regions in order to explore their environment more efficiently.

Regarding the different models that we have tried for *f* (*α*), the functions that reproduce the experimental data better are the power-like ones. Linear functions of the angle do not agree well with experiments; all reasonable candidates have been highly non-linear functions. Therefore, the larval behaviour reveals a complex and rich neural network behind the process of taking decisions, which works on a non-linear function, which is a common feature to neural circuits organized in layers^26^.

So far, the model does not take into account the larval dimensions. However, it would be possible to add terms to the weights *W* (***r*** → ***r***′) in eq. (3) to take into account the size of the larva, for example, by artificially increasing the value of these weights when two larvae would overlap on the new position ***r***′. In this case, we could take into account the fact that they cannot go to places already occupied by other larvae and even the attraction or repulsion between individuals could be modelled^27,28^. This would open the possibility to study simulated group behaviour, although at a higher computational cost.

An additional feature of larval taxis, studied for chemotaxis, is *weathervaning*, which is defined as miniature head-sweeping during runs resulting in curved tracks^29^. Whether *weathervaning* plays a role in phototaxis or not remains unclear. Since our statistical model is based on the local values of weights for the underlying Markov chain, *weathervaning* is not directly taken into account. The model is based on larval runs and turns with a single underlying mechanism governed by the weights in eq. (3). Moreover, Davies *et al*.^30^ have studied *weathervaning* for larval chemotaxis to conclude that it is the least crucial navigational parameter according to their model.

### Relevance for the neuronal network involved in larval phototaxis

Statistical models of larval navigation provide the first step towards understanding the underlying mechanisms that operate in the larval neuronal network to lead to decision-making. Next steps in understanding the neuronal basis of visual navigation may include to combine current information of the connectome with behavioural data and to correspondingly adapt a mathematical model^31,32^. This generalized Metropolis-Hastings-based model could also be used for other stimuli, the only requirement being that the intensities and gradients of these stimuli should be measured and quantified properly to define the details of the weights in eq. (3). Once these weights have been found for other stimuli, multi-sensory experiments could be carried out to see if these factors are additive towards larval navigation as suggested by Gepner *et al*.^12^.

## Materials and Methods

### Fly strains

Wild-type Canton S (WTCS) *D*. *melanogaster* larvae (courtesy of R. Stocker), and *glass*^*60j*^ mutants (Bloomington 509) were used for these experiments. All the fly stocks were kept at 25 °C in a 12-hour light-dark cycle. The stocks were fed with a conventional cornmeal medium containing molasses, fructose and yeast.

### Behavioural experiments

Larvae were selected for experiments after four days since the egg-laying of the parental flies, ensuring that they would correspond to the 3^rd^ larval stage (L3). Larvae were kept for at least 10 minutes in the dark with food before the phototaxis experiments were carried out. Thirty larvae were isolated from the food for each experiment and placed in water droplets with a paintbrush. The maximum time for the larval selection was 10 minutes and it was done under red light conditions. The larvae were left in the agarose plate without food and their tracks were recorded for 11 minutes. The first minute was not taken into account to let the larvae get used to the new conditions. The behaviour experiments were always carried out within the larval 12 light-hours.

### Tracking system

The experimental setup for all the experiments carried out with the projector forming a 40° angle (f1–f6 and Tilted, Pos and Neg) consists of a 23 × 23 cm agarose plate where the larvae can move freely (Fig. 1a). Larval movements were recorded with a Basler acA2500-14 gm camera equipped with a 1:14/12.5 mm Fujinon lens and placed directly above the tracking arena. The lens was incorporated with a red filter (635 nm, Qualimatest SA, Geneva, Switzerland). The agarose plate was illuminated with red LEDs that do not influence larval behaviour but enable the image recollection with the camera (Fig. 1a).

For the experiments f1–f6 and Tilted, Pos and Neg, an EB U04 projector was located at *x* = 35.5 cm, *y* = 0 cm, *z* = 28 cm. The projector was equipped with a 2″ Square BG40 coloured glass bandpass filter 335–610 nm and was placed forming a 40° angle with respect to the *x* − *y* plane formed by the agarose plate (zenithal angle, *θ*, Fig. 1a).

The experiments where the projector formed a 90° angle (D90-f1, D90-f2 and D90-f3) were carried out using a FIM table^33^, where we placed a 32 × 32 cm agarose plate. An ac2500-14 gm Basler camera with a 1:1.4/12.5 mm FUJINON lens was used to image. The lens was equipped with an LP 830 Near-IR longpass filter. The FIM table has 24 infra red LEDs on each side of the table to enable image acquisition with the camera due to the Frustrated Total Internal Reflection (FTIR) principle.

For these experiments, an Optoma X600 projector was used and it was placed forming a 90° angle (zenithal angle, Fig. 3a) with the agarose plate. The projector was located at *x* = −11.5 cm, *y* = 0 cm, *z* = 80 cm. Same as before, a 2″ Square BG40 coloured glass 335–610 nm bandpass filter was placed in front of the projector.

The custom-made LabView software^3,34^ was used to record the larval movies.

### Light intensity measurement on the agarose plate

Different light patterns were projected to obtain the different experimental scenarios against which the simulations could be validated. The intensity field varied in a different way in all of them. f1–f6 were uniform filters where the intensity variation was merely due to variation of the photon flux with the distance to the projector (Figs 1b and S1). In the patterns “Pos”, “Neg”, and “Tilted” used to explore directionality, an artificial modulation in the light gradient along the *x* or *y* directions was introduced and therefore there was a steeper change in light intensity. In “Pos”, the maximum light intensity was closer to the light source, same as in all the f1–f6 patterns, but the light intensity decreased along the −*x* axis with a gradient that was about 5 times steeper (Supplementary Tables S2 and S4). “Neg” was a 180° rotation of “Pos”; therefore, the intensity field decreased along the +*x* axis and the brightest area was located further away from the light source. “Tilted” was a 90° rotated version of the “Pos” pattern. In this case, the light gradient artificially varied along the y direction, therefore the intensity field decreased along the −*y* axis (Figs 2a and S3c).

Light intensities created by the different projected patterns were measured on the agarose plate using an *Ocean Optics USB400* spectrometer. Three equally-spaced points along the *x* axis (*y* = 0) were measured for the filters used to study light intensity (f1-f6, Supplementary Fig. S1) and nine points equally covering the *x* and *y* directions for the patterns related to directionality (“Pos”, “Neg”, and “Tilted”, Supplementary Fig. S3). Several measurements were taken for each point on different days and standard errors were calculated. The total intensity (W/m^2^) was obtained by integrating these spectra between 380 and 570 nm to include the blue and green wavelength regions of the spectrum relevant for Rh5 and Rh6 absorption spectra, but to exclude the red one (Supplementary Figs S1 and S3). Integrals have been performed by first defining an interpolating polynomial going through all the experimental points, and then using an accurate Gaussian-Konrod rule for integration^35^.

The expected variation of light intensity on the plate by a uniform source of light is described by assuming a steady rate of generation of photons. For the actual parameters of the geometrical setup, this has the implication of an approximate linear variation, which has been corroborated by measuring intensities on the plate (Supplementary Figs S1 and S3). Therefore, the spatial variation of intensities has been represented by a linear fit with

5 $$I(x,y)={a}_{0}+{a}_{1x}+{a}_{1y}$$

where *a*_0_ is the value at *x* = *y* = 0 and *a*_1*x*_, *a*_1*y*_ are the slopes along the *x* and *y* directions. Values for these coefficients are given in Supplementary Tables S2 and S4.

The air conditioning was turned on at 25 °C during the experiments to ensure a constant temperature in the agarose plate. Measurements of the temperature on the plate always yielded temperatures in the interval between 25 °C and 26 °C.

### Tracking data analysis

The acquired images of the larval tracks were analysed with the MAGAT Analyzer^3^. The features of each larva (head, tail and midline) were extracted from the videos and these data were analysed using a custom-made software written in MATLAB^36^.

Statistical analysis of the data was calculated using the Welch’s unpaired t-test to compare results with different genotypes and a regular unpaired t-test was used to compare larvae with the same genotype. The Benjamini-Hochberg procedure was applied to correct for multiple comparison. The statistical difference of results compared with zero was calculated using a one-sample t-test.

### Generalized Metropolis-Hastings chains

In our simulations, we assign transition probabilities between states in the Markov chain according to the Boltzmann distribution. Probabilities are assigned in the following way^37^:

1. *We keep a description of possible system configurations and the options presented to the system*. *These are determined by giving*:

- The initial position of the larvae, ***r*** = (*x*, *y*) and the final attempted position, ***r***′ = (*x*′, *y*′)*, where x*′ = *x* + Δ*x* *and y*′ = *y* + Δ*y*
- The light intensity at both points: *I*(***r***) *and I*(***r***′)

2. *A generator of random changes in the configurations*. We chose the next position using two independent Gaussian deviates with zero mean ($\bar{{\rm{\Delta }}}=0$) and standard deviation one (*σ* = 1) for the independent increments in the *x* and the *y* direction, Δ*x* and Δ*y* respectively. This defines a discrete-time continuous-space Markov chain of transition kernel ${k}_{0}({\boldsymbol{r}},{\boldsymbol{r}}^{\prime} )={e}^{-\frac{\parallel {\boldsymbol{r}}^{\prime} -{\boldsymbol{r}}{\parallel }^{2}}{2}}$.
  The standard deviation sets up a length scale that we adjust to the observation that the larvae approximately advance a distance equivalent to the length of its body in about ten moves. Therefore, the standard deviation is equivalent to approximately 0.1 mm.
3. *The larval local moves are described by a discrete-time continuous-space Markov chain of transition Kernel*:

6 $$k({\boldsymbol{r}}\to {\boldsymbol{r}}^{\prime} )={k}_{0}({\boldsymbol{r}}\to {\boldsymbol{r}}^{\prime} )\times {e}^{-\frac{W({\boldsymbol{r}}\to {\boldsymbol{r}}^{\prime} )}{T}}$$

where *W*(***r*** → ***r***′) = Δ*I*(***r*** → ***r***′) + *β* < *I* > *f*(*α*(***r*** → ***r***′)) (Eqs 3 and 4). Algorithmically, the new ***r***′ is chosen as follows:

- Choose ***r***′ according to the Gaussian model (*k*_0_), ***r***′ = ***r*** + *μ*, where *μ* is a bivariate Gaussian deviate with zero mean ($\bar{{\rm{\Delta }}}=0$) and standard deviation (*σ* = 1) in both dimensions.
- If *W*(***r*** → ***r***′) < 0, then ***r***′ is accepted with probability *P* = 1
- If *W*(***r*** → ***r***′) > 0, then ***r***′ is accepted with probability $P={e}^{-\frac{W({\boldsymbol{r}}\to {\boldsymbol{r}}^{\prime} )}{T}}$

4. *A control parameter T*. This parameter controls the weights so that the simulation can reproduce the experimental navigation index for a given light intensity pattern. *T* has irradiance units, same as *W*, and in a thermodynamics system it would be the equilibrium temperature. High values of *T* leads to small values of the argument in the exponential weight ${e}^{-\frac{W({\boldsymbol{r}}\to {\boldsymbol{r}}^{\prime} )}{T}}$, and the related transition step will occur with a probability near to 1 (*W* > 0).

The experimental recording stops tracking larvae that hit the border of the agarose plate. Accordingly, we introduce a similar boundary condition in our simulations and we stop tracking larvae that after *N* accepted steps have reached a distance to the origin greater or equal to 1150 *σ* ≈ 11.5 cm. Depending on the illumination conditions, this usually happens after a few thousands accepted steps. At that point, the Markov chain has an absorbing state. We have checked that the averaged *NI* and the angular probability distributions reach a quasi-stationary state before the experiment is terminated. For a given light pattern, each simulation was carried out with 30 individual larvae, and the average *NI* and its standard error have also been computed for a 30 larvae ensemble, making sure that the given *NI* is statistically significant.

### Determination of *f*(*α*)

The angular part in eq. (3) has been modelled to take into account the experimental angular distributions. The angle *α* is measured with respect to the *x* axis, being 0° the direction towards the projector and 180° the direction away from it (Fig. 4a). Two types of models for *f* (*α*) were tried: power-like models proportional to *α*^*n*^ and models based on *cos*^*n*^ (*α*), taking into account that cos(*α*) = Δ*x*/Δ*l* (Supplementary Fig. S8). Each model was assessed calculating the standard deviation of the angular probability distribution of the simulated paths compared to the experimental ones. Both the experimental and simulated paths were binned in 30° angles and the probabilities for both experimental and simulated cases were compared. The best fit to experiments across all the different projected patterns was found for f(*α*) = 1 − (*α*/180)^4^, as shown in Supplementary Table S7, where we give the root-mean-squared (RMS) deviation between experimental and simulated angular distributions for all models tried for *f* (*α*).

### Determination of *β*

The value for the parameter *β* (*β* = 1.4/100) was determined by taking the “Tilted” pattern as a case where the two terms of the objective function (intensity and directionality) are most decoupled. Consequently, first the *NI*_*y*_ in the “Tilted” pattern was simulated assuming that only the first term in the objective function would exist. That procedure yields a value for the effective *T.* Afterwards, the experimental value for *NI*_*x*_ was used to find a value for the parameter *β*. As a final consistency check, both the *NI*_*y*_ and the *NI*_*x*_ were simultaneously recalculated using the two parts of the objective function, obtaining a refined value for *T* that fits the two available experimental values at the same time.

### Data availability

The datasets generated and analysed furing the current study are available from the corresponding author upon request.

## Electronic supplementary material

Supplementary figures
