# Supplementary material for: Statistical modelling of navigational decisions based on intensity versus directionality in Drosophila larval phototaxis
Source: Sci Rep. 2018 Jul 26;8:11272. doi: 10.1038/s41598-018-29533-0 (PMC6062584; doi:10.1038/s41598-018-29533-0)
Supplement: Supplementary file 1 — Supplementary figures [file 41598_2018_29533_MOESM1_ESM.pdf]

Supplementary Figures

Statistical modelling of navigational decisions based on intensity versus directionality in *Drosophila* larval phototaxis

Lucia de Andres-Bragado[1], Christian Mazza[2*], Walter Senn[3*] and Simon G. Sprecher[1*]

[1]Department of Biology, University of Fribourg, Fribourg, Switzerland

[2]Department of Mathematics, University of Fribourg, Fribourg, Switzerland

[3]Department of Physiology, University of Bern, Bern, Switzerland

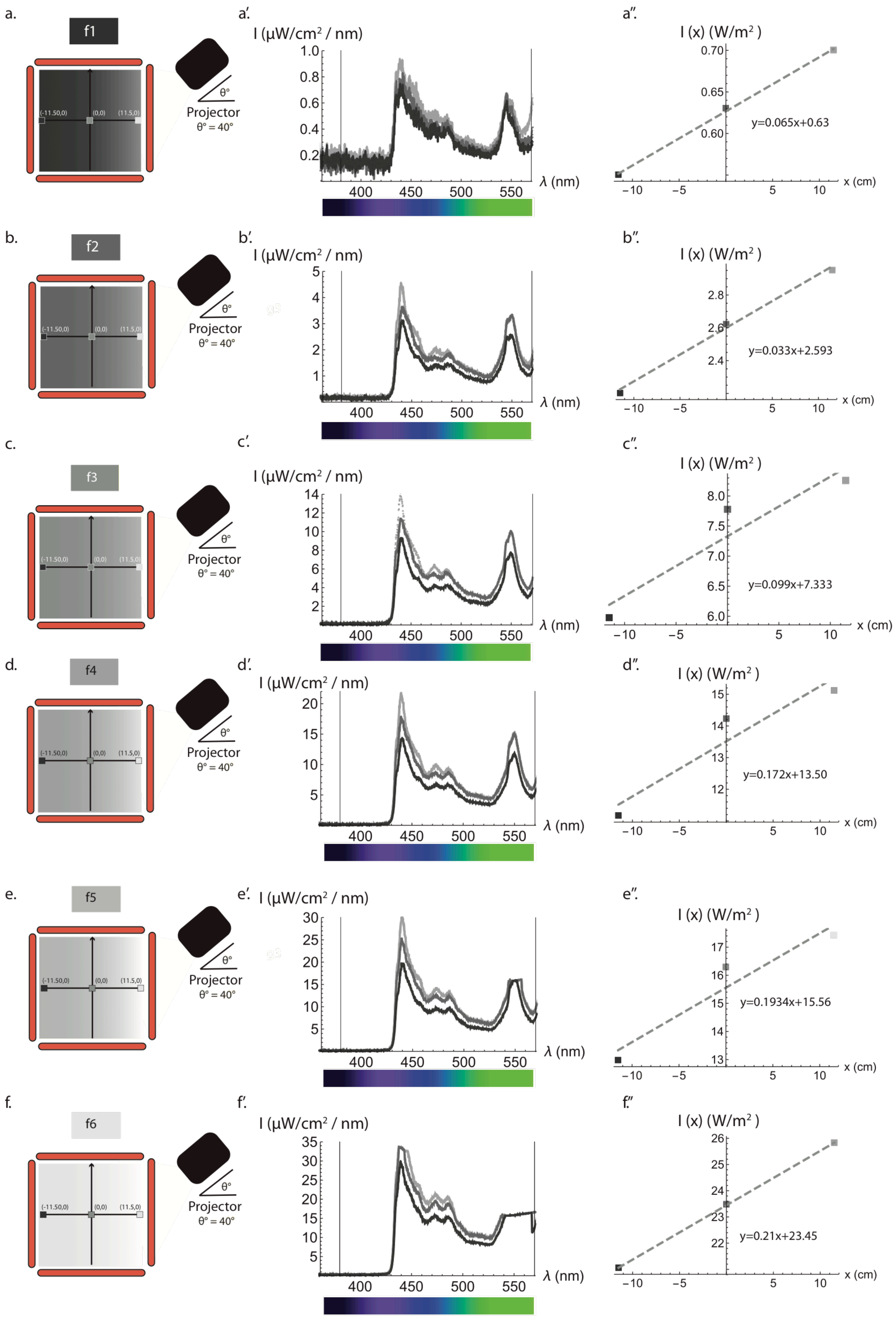
a.
f1
Projector
θ° = 40°
a'.
I (μW/cm² / nm)
λ (nm)
a''.
I (x) (W/m²)
y=0.065x+0.63
x (cm)
b.
f2
b'.
b''.
y=0.033x+2.593
c.
f3
c'.
c''.
y=0.099x+7.333
d.
f4
d'.
d''.
y=0.172x+13.50
e.
f5
e'.
e''.
y=0.1934x+15.56
f.
f6
f'.
f''.
y=0.21x+23.45

Supplementary Figure S1. **Light spectrum along the agarose plate for the f1 – f6 filters**. (**a**-**f**) The light intensity was measured with an OceanOptics USB400 spectrophotometer in the agarose plate in three points for the f1 – f6 filters. Light intensities were measured for all the filters along the x axis of the agarose plate at three points: (1) one closer to the projector, at $(11.5,0)$ cm (white square), (2) in the centre of the agarose plate $(0,0)$ cm (grey square) and (3) in the point furthest away from the projector $(-11.5,0)$ cm (black square). Replicates of each of the measurements were taken on different days. (**a'**-**f'**) Light intensity (I) is given in μW/cm^2/nm as a function of the wavelength λ in nm. The light intensity measured on these three points was plotted and integrated between 380 nm and 570 nm (vertical lines), which is the biologically relevant wavelength range for the larvae. (**a''**-**f''**) The variation of light intensity along the x axis of the agarose plate was modelled with a linear regression for each of the filters.

**Filter**	**a_0**	**a_{1x}**
f1	0.627 ±0.002	0.0065 ±0.0002
f2	2.59 ±0.02	0.033 ±0.002
f3	7.3 ±0.3	0.1 ±0.03
f4	13.5 ±0.5	0.17 ±0.05
f5	15.6 ±0.5	0.19 ±0.05
f6	23.45 ±0.02	0.207±0.002

Supplementary Table S2. Light measurements for the f1 – f6 projected filters. The three measured points along the x axis of the agarose plate for filters f1 – f6 (Supplementary Figure S1 **a**-**f**) were used to model the variation with a linear regression. Light intensity is quantified with a least-square polynomial fit to intensities. The slope of the linear variation of intensities ($a_{1x}x$ in W/m^2/cm) and the intercept (a_0 in W/m^2) can be seen in this table for each of the filters.

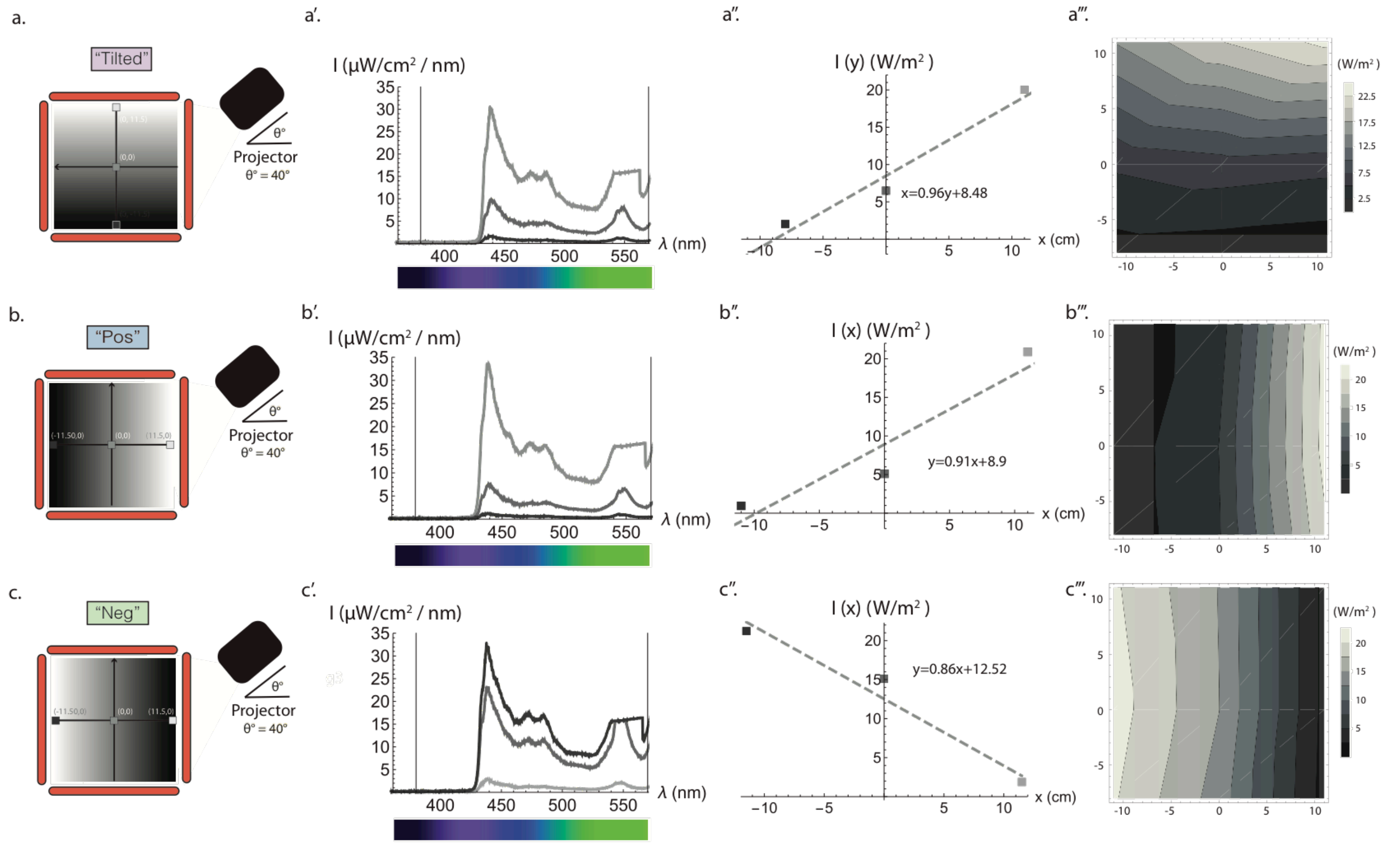

Supplementary Figure S3. **Light spectrum along the agarose plate for the directionality patterns.** (**a-c**) The light intensity was measured as in Supplementary Figure S1 for the "Tilted", "Pos" and "Neg" patterns in nine points forming a homogenous grid in x and y. The gradients of light intensities along the x axis and along the y axis were obtained from that grid. Replicates of each measurement were taken on different days. (**a'-c'**) Same as in Supplementary Figure S1 for "Tilted", "Pos", and "Neg". (**a''-c''**) Variation of light intensity along the the y axis on the agarose plate for "Tilted" and along the x axis for "Pos" and "Neg". A linear regression for each of the patterns was found to describe well the pattern of intensities. (**a'''-c'''**) Contour plot for each of the projected filters.

Filter	a_0	a_{1x}	a_{1y}
“Pos”	8.9 ± 2.7	0.9 ± 0.3	0.06 ± 0.04
“Neg”	12.5 ± 1.8	-0.9 ± 0.2	0.05 ± 0.04
“Tilted”	8.5 ± 1.5	0.02 ± 0.004	0.96 ± 0.2

Supplementary Table S4. Light measurements for the projected directionality patterns. Light intensity is quantified with a least-square polynomial fit to intensities, $a_0 + a_{1x}x + a_{1y}y$. This table shows the values for the linear regression parameters: the slope of the linear variation of the light intensity along the x axis (a_{1x} in W/m^2/cm), along the y axis (a_{1y} in W/m^2/cm) and the intercept (a_0 in W/m^2) for each of the projected patterns.

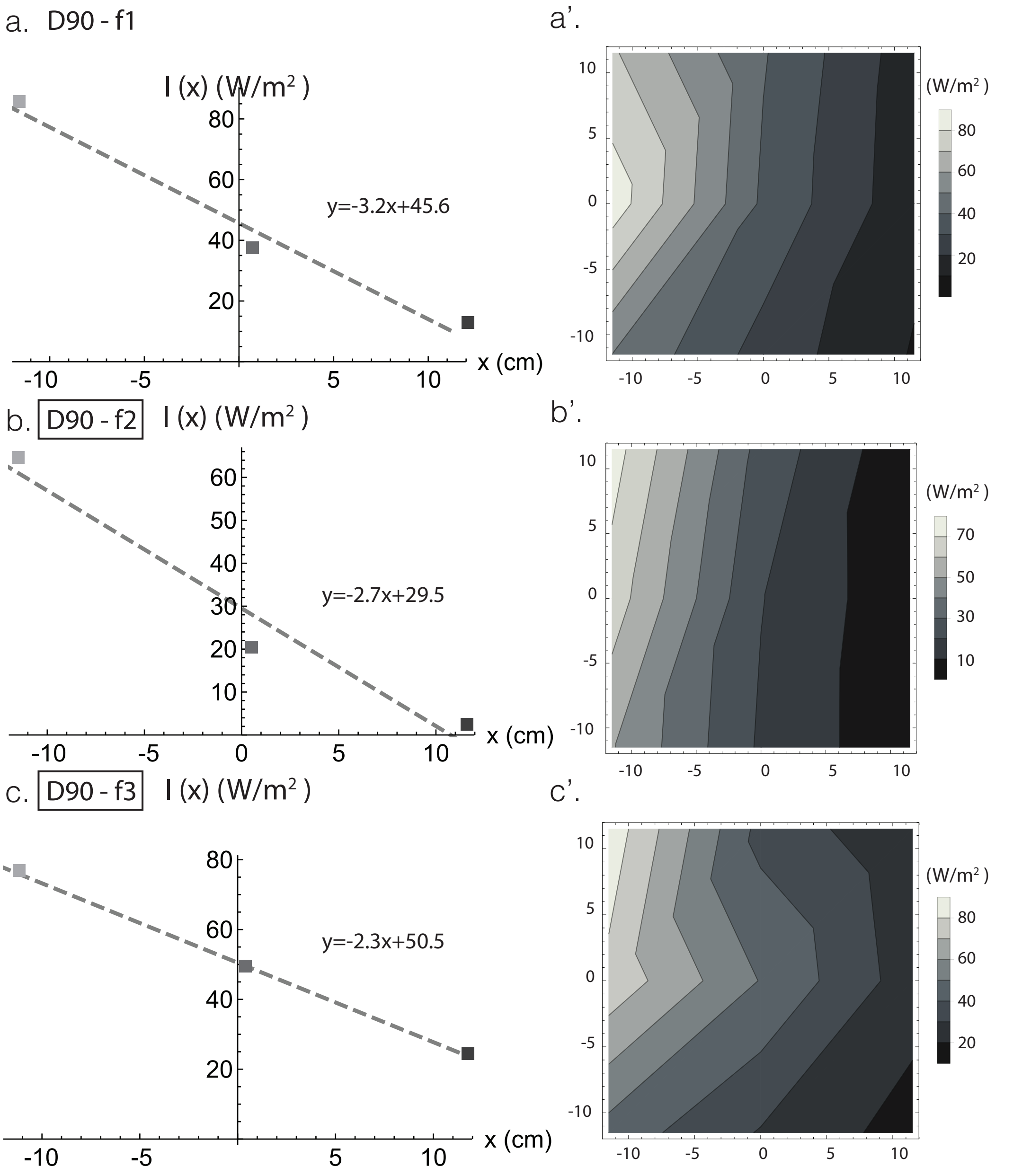

Supplementary Figure S5. **Light spectrum along the agarose plate for the D90-f1, D90-f2 and D90-f3 patterns.** (**a**-**c**) Same as in Supplementary Figure S1, the variation of light intensity along the agarose plate was described with a linear regression, calculated from light intensity measurements taken in nine points of the agarose plate. (**a'**-**c'**) Contour plot for each of the projected filters.

**Filter**	**a_0**	**a_{1x}**	**a_{1y}**
D90-f1	45.6 ± 5.41	3.2 ± 0.57	0.64 ± 0.23
D90-f2	29.5 ± 6.39	2.7 ± 0.68	0.34 ± 0.09
D90-f3	50.5 ± 0.69	2.3 ± 0.07	0.32 ± 0.83

Supplementary Table S6. Light measurements for the D90-f1, D90-f2 and D90-f3 projected filters. The change of light intensity along the agarose plate was quantified with a least-square polynomial, $a_0 + a_{1x}x + a_{1y}y$ and the variation was modelled with a linear regression. This table shows the values for the slope of the linear variation along the x axis (a_{1x} in W/m^2/cm), along the y axis (a_{1y} in W/m^2/cm) and the intercept (a_0 in W/m^2) for the three projected patterns with the projector forming a 90-degree angle with the agarose plate: D90-f1, D90-f2 and D90-f3.

Filter	$\boldsymbol{\alpha^2}$	$\boldsymbol{\alpha^4}$	$\boldsymbol{\alpha^6}$	$\boldsymbol{cos(\alpha)}$	$\boldsymbol{1+cos(\alpha)}$	$\boldsymbol{\sqrt{1+cos(\alpha)}}$
f1	0.013	0.013	0.012	0.015	0.014	0.013
f2	0.009	0.008	0.007	0.014	0.011	0.009
f3	0.064	0.059	0.057	0.073	0.068	0.064
f4	0.009	0.006	0.009	0.024	0.015	0.0102
f5	0.018	0.027	0.034	0.024	0.015	0.0319
f6	0.011	0.008	0.013	0.030	0.019	0.013
Average RMS	0.021	0.020	0.022	0.029	0.024	0.024

Supplementary Table S7. Comparison of the different models for $\mathbf{f(\alpha)}$. Simulations were carried out using different models for $f(\alpha)$ and tested against the f1 – f6 filters. Simulations for each model were carried out 30 times and the experimental ones were calculated doing 10 experiments with around 30 larvae each. Both the experimental and simulated angular probability distributions were binned in 30° angles. Each model was assessed by calculating the root mean squared deviation (RMS) between the experimental and simulated angular probability distributions for the different binned angles from $0°$ to $180°$. Then, the average of the RMS for all the filters was compared for each model of $f(\alpha)$. The smallest overall RMS was obtained with $f(\alpha) \propto 1 - \alpha^4$.

a.

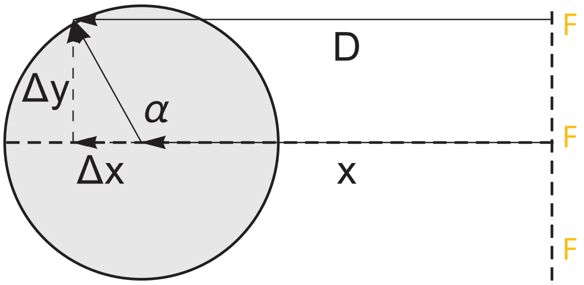

Supplementary Figure S8. Geometrical diagram for the directionality part of the cost function. The source of light is approximated by a plane, F. The light came from right $(+x)$ to left $(-x)$. The angular distribution is a function of the angle of the direction of the larva, α, which is a function of the displacement $\alpha = \arctan\frac{\Delta y}{\Delta x}$. The increment in the distance to the plane F depends on the displacement $\Delta x = \Delta l\, cos\alpha$, where $\Delta l = \sqrt{\Delta x^2 + \Delta y^2}$.
